# Topological materials for full-vector elastic waves

**DOI:** 10.1093/nsr/nwac203

**Published:** 2022-09-24

**Authors:** Ying Wu, Jiuyang Lu, Xueqin Huang, Yating Yang, Li Luo, Linyun Yang, Feng Li, Weiyin Deng, Zhengyou Liu

**Affiliations:** School of Physics and Optoelectronics and State Key Laboratory of Luminescent Materials and Devices, South China University of Technology, Guangzhou510640, China; Institute of Solid Mechanics, Midea Corporate Research Center, Midea Group, Foshan528311, China; School of Physics and Optoelectronics and State Key Laboratory of Luminescent Materials and Devices, South China University of Technology, Guangzhou510640, China; School of Physics and Optoelectronics and State Key Laboratory of Luminescent Materials and Devices, South China University of Technology, Guangzhou510640, China; School of Physics and Optoelectronics and State Key Laboratory of Luminescent Materials and Devices, South China University of Technology, Guangzhou510640, China; School of Physics and Optoelectronics and State Key Laboratory of Luminescent Materials and Devices, South China University of Technology, Guangzhou510640, China; Department of Astronautic Science and Mechanics, Harbin Institute of Technology, Harbin150001, China; Centre for Quantum Physics, Key Laboratory of Advanced Optoelectronic Quantum Architecture and Measurement (MOE), School of Physics, Beijing Institute of Technology, Beijing100081, China; School of Physics and Optoelectronics and State Key Laboratory of Luminescent Materials and Devices, South China University of Technology, Guangzhou510640, China; Key Laboratory of Artificial Micro- and Nanostructures of Ministry of Education and School of Physics and Technology, Wuhan University, Wuhan430072, China; Institute for Advanced Studies, Wuhan University, Wuhan430072, China

**Keywords:** topological insulators, elastic waves, edge states

## Abstract

Elastic wave manipulation is important in a wide variety of applications, including information processing in small elastic devices and noise control in large solid structures. The recent emergence of topological materials has opened new avenues for modulating elastic waves in solids. However, because of the full-vector feature and the complicated couplings of the longitudinal and transverse components of elastic waves, manipulating elastic waves is generally difficult compared with manipulating acoustic waves (scalar waves) and electromagnetic waves (vectorial waves but transverse only). To date, topological materials, including insulators and semimetals, have been used for acoustic and electromagnetic waves. Although topological materials with elastic waves have also been reported, the observed topological edge modes lie on the domain wall. A natural question arises: Is there an elastic metamaterial with topological edge modes on its own boundary? Here, we report a 3D metal-printed bilayer metamaterial that topologically insulates elastic waves. By introducing chiral interlayer couplings, the spin–orbit couplings for elastic waves are induced, which give rise to nontrivial topological properties. Helical edge states with vortex features were demonstrated on the boundary of the single topological phase. We further show a heterostructure of the metamaterial that exhibits tunable edge transport. Our findings could be used in devices based on elastic waves in solids.

## Teaser

Topological elastic metamaterial was realized in a 3D metal-printed bilayer structure, enabling helical edge states of full-vector elastic waves on the boundaries of a single phase.

## INTRODUCTION

Elastic metamaterials (EMMs), which are artificially designed structures [1–3], are widely used to manipulate elastic waves in ways that are not found in nature, such as non-destructive testing [4], wave guiding [5] and information processing [6]. However, wave transport in conventional EMMs suffers from unavoidable backscattering in the presence of bending and defects. Recently, extensive efforts have been made to explore topological EMMs [7,8], which possess high-efficiency transport of boundary modes or higher-order corner states, following the discovery of topological insulators in condensed-matter physics [9–12].

In two dimensions, there are two types of topological EMMs for the conventional or first-order topological insulator phase. The first hosts chiral edge states on its own boundary, in which the piezoelectric units break the time-reversal symmetry [13,14], mimicking a quantum anomalous Hall insulator. However, the introduction of active components drastically increases engineering complexity. Analogizing the quantum spin (valley) Hall insulator, the other possesses helical boundary states with time-reversal symmetry and has been realized in multi-scale EMMs [15–22]. Note that the full-vector feature of the elastic wave equation has yet to be fully considered in these systems. Boundary states are usually localized at the domain walls between two distinct topological phases, acting as interface states. It remains an open question whether an EMM in the continuum can host the topological boundary states localized at the boundary of a single phase, i.e. the edge states.

Here, we experimentally realize a topological EMM that inherently preserves helical edge states in a bilayer structure. Benefitting from the rich and flexible interlayer couplings, bilayer structures provide new routes for exploring the topological phase of matter [23–27]. Inspired by these works, we constructed a bilayer structure that has chiral interlayer coupling to induce spin–orbit coupling for full-vector elastic waves and gives rise to nontrivial topological properties. Owing to advanced 3D metal-printing technology [28], an EMM with chiral interlayer coupling can be fabricated using stainless steel with high reliability. This topological EMM provides a low-dissipation platform for elastic wave manipulation.

## RESULTS

### Bulk topological property

**Figure 1.**
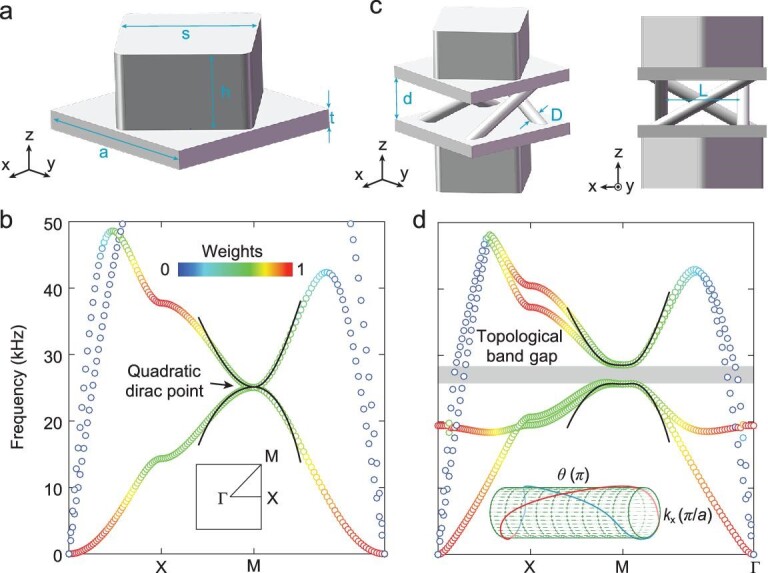
Bulk dispersions of the single-layer and bilayer EMMs. (a) Unit cell of the single-layer EMM. (b) Bulk dispersion of the single-layer EMM. A quadratic degeneracy is preserved at the ${\rm{M}}$ point. The inset shows the first Brillouin zone. (c) and (d) Unit cell and bulk dispersion of the bilayer EMM. Chiral interlayer coupling is introduced by four tilted pillars, which opens a topologically nontrivial band gap (the grey region in (d)). In (b) and (d), the colour maps represent the proportion of the out-of-plane displacement $| w |$ and the black solid curves denote the fitting data of the effective Hamiltonians. Inset: the non-Abelian Wilson loop. The geometry parameters are: lattice constant $a = 10.0{\rm{\ mm}}$, plate thickness $t = 1.0{\rm{\ mm}}$, length (height) of square block $s = 7.0{\rm{\ mm}}$ ($h = 4.0{\rm{\ mm}}$), distance between two layers $d = 3.5{\rm{\ mm}}$, diameter of the tilted pillar $D = 1.0{\rm{\ mm}}$ and distance between two opposite titled pillars $L = 6.4{\rm{\ mm}}$.

We begin with a single-layer EMM of a square lattice. As shown in Fig. 1a, a square block was located at the centre of the plate to modulate the elastic waves, giving rise to the band structure (Fig. 1b). Protected by the symmetry of the ${C}_{4{\rm{v}}}$ point group, double degeneracy occurs determinately at the ${\rm{M}}$ point. Degeneracy is a typical quadratic Dirac point, featured by quadratic dispersions with opposite curvatures and $2\pi $ Berry phase around the point. The realization of topological insulator phases in a single EMM requires the quadratic Dirac degeneracy to be lifted by breaking parity or time-reversal symmetries [29]. For a single-layer EMM, breaking parity symmetry hardly opens a direct band gap at the ${\rm{M}}$ point, whereas breaking time-reversal symmetry requires active components, which makes fabrication difficult.

We introduce interlayer coupling between two identical single-layer EMMs. Without coupling, the bilayer EMM has a copy of the quadratic degeneracy at the ${\rm{M}}$ point. To open a band gap at that point, as depicted in Fig. 1c, chiral interlayer couplings are introduced by four tilted pillars, which break the parity symmetry and give rise to a topological EMM phase. A thorough description of the ${\rm{M}}$ point can be obtained using an effective Hamiltonian deduced from the $k \cdot p$ perturbation theory. Considering all crystalline symmetries, the linear part of the perturbation Hamiltonian vanishes and the explicit form of the quadratic part is strictly constrained (see Supplementary Note 1), leading to the perturbation Hamiltonian as:


(1) \begin{eqnarray*} \delta H &=& \left( {\Delta k_x^2 + \Delta k_y^2} \right) {q}_0 + 2\Delta {k}_x\Delta {k}_y{q}_1{\sigma }_x \\ && +\, \left( {\Delta k_x^2 - \Delta k_y^2} \right){q}_2{\sigma }_z + \eta {\tau }_y{\sigma }_y. \end{eqnarray*}

Here, the Pauli matrices ${\tau }_i$ and ${\sigma }_i$ denote the layer pseudospin and the basis eigenmodes constituting the quadratic degeneracy in a single-layer EMM, respectively; $({\rm{\Delta }}{k}_x,{\rm{\Delta }}{k}_y)$ is the dimensionless wavevector deviating from the ${\rm{M}}$ point; and ${q}_i\ ( {i\ = \ 0,1,2} )$ and $\eta $ represent the strengths of the intralayer and interlayer couplings, respectively.

The perturbation Hamiltonian, which is consistent with the symmetries of the system, is capable of describing the band structures of the bilayer EMM. For $\eta = 0$, the perturbation Hamiltonian in Equation (1) describes a single-layer case. By fitting the dispersions around the ${\rm{M}}$ point of the single-layer EMM, ${q}_i$ are determined as ${q}_0 = 0.38{\rm{\ H}}{{\rm{z}}}^2$, ${q}_1 = 1.26{\rm{\ H}}{{\rm{z}}}^2$ and ${q}_2 = 1.39{\rm{\ H}}{{\rm{z}}}^2$. The fitting dispersions are plotted as black solid lines in Fig. 1b, showing consistency with the simulated ones (hollow circles). When $\eta \ne 0$, the last term in Equation (1), representing the coupling between the layer pseudospins and the eigenmodes of a single layer, gives rise to synthetic spin–orbit coupling for the bilayer EMM and produces a band gap at the ${\rm{M}}$ point (grey region in Fig. 1d). Determined by the width of the bandgap, $\eta $ is equal to $1.39{\rm{\ H}}{{\rm{z}}}^2$. With ${q}_i$ and $\eta $, in the vicinity of the ${\rm{M}}$ point, the fitting curves (black solid lines in Fig. 1d) obtained using Equation (1) capture well with the simulated dispersions for the bilayer EMM (hollow circles). The colours of the simulated dispersions (Fig. 1b and d) represent the weight of the out-of-plane polarization with respect to the total polarization (see ‘Methods’ section). This shows that near the ${\rm{M}}$ point, the modes with different polarizations hybridize and polarize partly in the out-of-plane direction. In the frequency range of the bandgap, the modes near the ${\rm{\Gamma }}$ point were hardly disturbed and retained their in-plane features. This facilitates experimental measurements by detecting the out-of-plane polarization in the frequencies of the band gap, where the distractions of modes far away from the ${\rm{M}}$ point are automatically blocked.

While the elastic waves in the bilayer EMM are vectorial in nature, the EMM cannot be described by an equation that involves only out-of-plane polarization but includes in-plane polarization. In fact, all the three polarizations, i.e. the longitudinal mode and the shear horizontal and vertical modes, have been introduced to construct the topology for our system. These full-vector properties are essential for the effective Hamiltonian in Equation (1), from which the topological properties of the bilayer EMM can be deduced analytically. After a similar transformation of a unitary matrix $U = - \frac{1}{2}( _{1}^{1}\quad _{\,i}^{- i})\,\,\otimes\,\,( _{i}^{1}\quad _{1}^{i})$, the Hamiltonian turns into a block-diagonal form $\delta H^{\prime} = U\delta H\ {U}^{ - 1} = {H}_ \uparrow {\rm{\ }} \oplus {H}_ \downarrow $, where the block matrices ${H}_{ \uparrow / \downarrow } = ( {\Delta k_x^2 + \Delta k_y^2} )\ {q}_0 + 2\Delta {k}_x\Delta {k}_y{q}_1{\sigma }_x + ( {\Delta k_x^2 - \Delta k_y^2} ){q}_2{\sigma }_y \pm \eta {\sigma }_z$ represent the pseudospin up/down Hamiltonian. The two-block matrices share an identical bulk gap for nonzero $\eta $ and opposite Chern numbers, ${C}_{ \uparrow / \downarrow } = \pm {\rm{sgn}}( \eta )$ for the first bulk band. A numerical approach to topological properties can be performed in parallel by calculating the non-Abelian Wilson loop [30] based on the simulated data (see Supplementary Note 2), as shown in the inset of Fig. 1d, which again demonstrates the nontrivial topology of the bilayer EMM.

### Topological edge states

**Figure 2.**
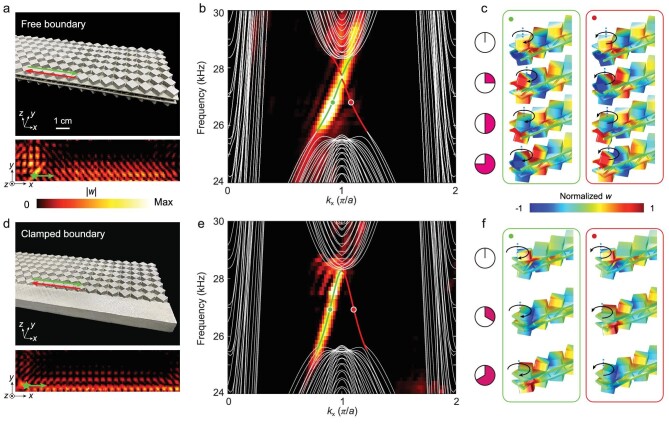
Measured helical edge states on the free and clamped boundaries. (a) Upper panel: photograph of the 3D metal-printing sample for the free boundary. Lower panel: Measured distribution of $| w |$ along the free boundary. Green star: a point source. (b) Measured (colour map) and simulated (curves) dispersions projected on the free boundary. The red and green curves denote dispersions of the edge states counter-propagating along the free boundary (illustrated by red and green arrows in (a)). (c) Simulated field distributions of the edge states, marked by the circles in (b), at sequential instants. The black arrows indicate the rotation of the square blocks around their centres (blue dashed lines). (d)–(f) The same as in (a)–(c), but for the clamped boundary.

With topologically nontrivial bulk properties, topological edge states are expected at the sample boundaries, regardless of whether they are free or clamped. In contrast to the previously proposed topological boundary states for elastic waves, which propagate along the interface between two domains, the edge states require only a single domain. We fabricated two samples with free and clamped boundaries, as shown in Fig. 2a and d, each of which was composed of $30 \times 7$ unit cells (see ‘Methods’ section and Supplementary Note 3). For convenience and efficiency, we excited the flexural motion using a piezoelectric actuator, which was glued at the free or clamped boundaries of the sample (denoted by the green stars). Undoubtedly, edge states can be excited by a source with in-plane vibrations (see Supplementary Note 5). In the experiments, we detected the out-of-plane displacement fields (*w*) using a vertical laser vibrometer (see ‘Methods’ section). The lower panels in Fig. 2a and d show that along the two types of boundaries, edge states are excited and propagate compactly along the boundaries at a frequency of $26.75\ {\rm{kHz}}$.

The experimental dispersions of the elastic edge states isolated from the bulk are shown in Fig. 2b and e. The colour maps show the experimental results and the solid curves represent the simulated results. The experimental and numerical results consistently showed the existence of topological edge states for the free boundary, as shown in Fig. 2b. We note that a tiny band gap exists at ${k}_x = \pi /a$, due to the coupling of the edge states with the up and down pseudospins at the same boundary. The band gap was too small ($0.3\% $ in the simulation) to be detected experimentally. For the clamped boundary condition, we obtain a pair of gapless edge states, denoted by the red and green curves in Fig. 2e, resulting in pseudospin up- and down-edge states counter-propagating along the boundaries. Note that only the edge-state dispersions with positive group velocities are measured because of the sources located on the left side. The simulated and measured results were in good agreement. Moreover, gapless helical edge states can also exist at the $45^\circ $ boundary, as illustrated in Supplementary Note 4.

The behaviour of pseudospin-momentum locking of the helical edge states is reflected by the profiles of their eigenmodes at both the free and clamped boundaries. The pseudospin is constructed by the layer degree of freedom and reflected by the vortices in the field distributions of the edge states. As shown in Fig. 2c and f, at different moments, the amplitudes of the vortices give rise to the rotations of the square blocks, whose directions lock to the edge-state propagations, i.e. forward or backward. Specifically, the edge states propagating forward (backward) host the vortex in the clockwise (anticlockwise) direction. Therefore, the topological edge states are locked in the vortex directions. We further utilized multiple sources with different phases to selectively excite the pseudospin up- and down-edge states (Supplementary Note 5).

**Figure 3.**
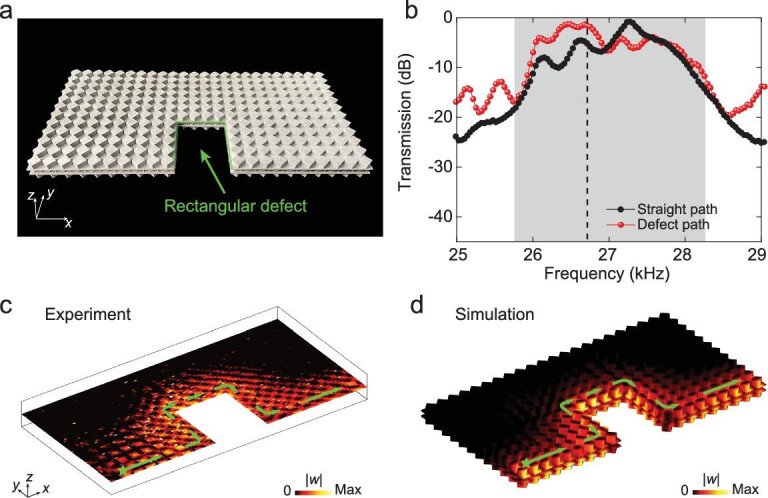
Robust transport of the elastic edge states in the presence of a rectangular defect. (a) Photograph of the EMM sample with a rectangular defect along the free boundary. (b) Measured transmission for the defect path (red dots) compared with that for a straight path (black dots). The grey region denotes the bulk band gap. (c) and (d) Measured and simulated distribution of $| w |$ at $26.75\ {\rm{kHz}}$, respectively. Green stars denote the sources.

One distinct feature of topological edge states is their robust one-way transport along boundaries, even with imperfections such as sharp corners. To investigate this, we designed a sample with a rectangular defect consisting of four $90^\circ $ angles (Fig. 3a). The source was located at one end of the free boundary and composed of $34$ unit cells. We excite chirped signals with frequencies ranging from $23.5\ {\rm{kHz}}\ $to $30.5\ {\rm{kHz}}$. In Fig. 3b, we compare the measured transmission of the defect path with that of a straight sample of the same length. The slight difference between these two transmission curves within the topological gap (grey region) demonstrates the weak backscattering of the edge state propagating along the rectangular defect. Fig. 3c and d shows the field strength $| w |$ at a frequency of $26.75\ {\rm{kHz}}$ (dashed line in Fig. 3b). The experimental and simulated results showed good agreement, confirming that the elastic wave propagated smoothly around the rectangular defect. The robust transport of the elastic waves along the clamped boundary is shown in Supplementary Note 5. In addition, it is worth pointing out that the 3D metal-printed samples [28] possess lower energy losses, advancing the design of new functional devices for elastic waves.

### Transport property of edge states

**Figure 4.**
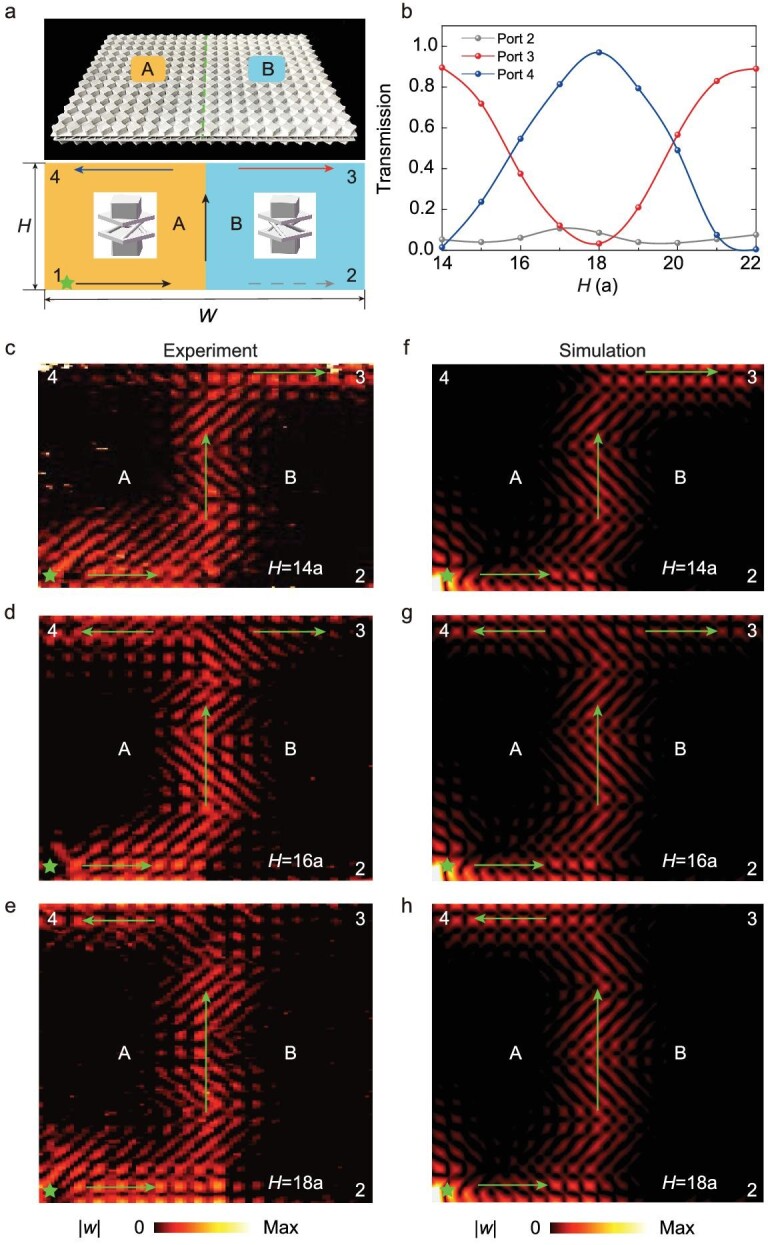
Selective transport at topological channels for elastic edge states. (a) Photograph of the sample, composed of A and B structures. There are four ports in the device, including one input, denoted as 1, and three output ports, denoted as 2, 3 and 4, respectively. The height and width of the sample are denoted as *H* and *W*. (b) Transmissions in output Ports 2–4 with respect to *H* at $26.75\ {\rm{kHz}}$. (c)–(h) Measured and simulated $| w |$ for $H = 14a$, $16a$ and $18a$, respectively. The green stars denote the point sources. The green arrows show the directions of edge-state propagation.

Beyond a single phase of EMM, we further demonstrate that the transport path can be selected by combining two topologically distinct EMMs, paving the way for exploring devices such as splitters and switches. From the top, the unit cell with an anticlockwise (clockwise) chiral interlayer coupling is denoted as A (B). It can be predicted that a doubled number of edge states will emerge at the interface between A and B, enabling the construction of complex networks for elastic wave manipulations (see Supplementary Note 6). As shown in Fig. 4a, we designed a topological device that includes four ports, formulated by A and B. The source was located at Port 1, marked by a green star, and the right and left sides of the device were set as absorbing boundaries to avoid reflections. The width of the device was $W = 20a$, while the height *H* could be manually tuned. We calculated the energy flow out of Ports 2–4, with height *H* ranging from $14a$ to $22a$, as shown in Fig. 4b. It is found that the transmissions of Ports 3 and 4 wane and wax with a period of $8a$ when the height changes. This oscillation feature originates from the interference of the two forward topological edge states belonging to A and B. Furthermore, because of spin-momentum locking, there is little energy flow to Port 2.

Finally, we fabricated three representative samples, $H = 14a$, $16a$ and $18a$, to demonstrate the above path-selection phenomenon in the experiments. We observed that for $H = 14a$, almost all of the elastic wave energy flowed to Port 3 (Fig. 4c). As the height increases to $H = 16a$, energy flows to Ports 3 and 4 (Fig. 4d). When $H = 18a$, nearly all of the energy flowed into Port 4 (Fig. 4e). The experimental fields showed good agreement with the simulations (Fig. 4f–h), demonstrating that the transport of the elastic wave can be adjusted by the height of the device. In addition to the free boundaries adopted here, samples with clamped boundaries are simulated in Supplementary Note 7 and show similar path-selection properties.

### CONCLUSION

In summary, we have proposed and realized a topological EMM possessing synthetic spin–orbit coupling that supports robust helical edge states. We experimentally demonstrate that topological modes are available for reflection immune propagation, as well as flexible tunability of elastic waves. It is noted that the previous work [7] theoretically proposes an EMM hosting edge states, but it remains a huge challenge to observe the edge states in experiments due to the tiny bandgap. We anticipate our bilayer EMM to be a versatile platform to fulfil the applications for both interface and surface waves, offering better flexibility for device realization. For example, the elastic edge states in EMM may find applications in splitters and switches in acoustic devices, enabling the construction of a monolithic elastic network. By stacking the structure layer by layer, our 2D topological EMM can be extended to 3D systems with intriguing topological transport, such as robust surface waves and higher-order hinge states [31,32]. Furthermore, the proposed configuration can also be regarded as a bulk structure for investigating topological defects [33–35].

## METHODS

### Numerical simulations

We used finite-element software to obtain all the simulations using COMSOL Multiphysics with the Solid Structure module. We performed an eigenfrequency study with a parameter sweep to calculate the band structures and projected dispersions. We simulated displacement fields with low-reflection boundary conditions based on a frequency-domain study. The largest and smallest element sizes were set to be $a/13$ and $a/20$, ensuring sufficient simulation accuracy. Each unit cell was meshed by 14 586 elements and totally has 99 159 degrees of freedom. For the material properties of 316L stainless steel, we chose Young's modulus $E = 190{\rm{\ Gpa}}$, Poisson's ratio $\mu = 0.26$ and mass density $\rho = 8000{\rm{\ kg}}/{{\rm{m}}}^3$.

### Sample preparation

We fabricated the samples using a 3D metal-printed method, specifically selective laser melting technology. By leveraging the focusing laser spot, the prefabricated metal powder was rapidly melted and our samples made of 316L stainless steel with sophisticated shapes could be obtained directly; the resulting density could reach >$99{\rm{\% }}$. After printing, we polished the surface and removed redundant supports, which were essential during the printing process (see Supplementary Note 2 for more details). The accuracy of this technology was ≤$20{\rm{\ \ \mu m}}$, which is suitable for our experimental samples.

### Experiments

For the topological edge-state measurements shown in Fig. 2, we used a piezoelectric ceramic exciter as a point source to stimulate elastic waves. Despite the stimulation with vibration in the out-of-plane direction, the excited waves contain both in-plane and out-of-plane components. We applied an ethylene–vinyl acetate copolymer to absorb extra elastic waves at the rest of the boundaries. We measured the frequency responses of 30 scanned unit cells along the clamped and free boundaries. The amplitudes and phases were obtained using a laser vibrometer combined with a network analyser. To obtain the dispersion curves of the boundary states, we measured the steady-state frequency response of the entire sample by spatial scanning with 1-mm steps (see Supplementary Note 2 for more details). We performed a fast Fourier transform of $| w |{\rm{exp}}( {i\varphi } )$ to plot the dispersion curves. To improve the resolution of the measured dispersions, the source was set at one side of the boundary; therefore, only the topological edge state with positive group velocity could be detected. Owing to the time-reversal symmetry, the edge state with a negative group velocity can be determined using mirror symmetry. We also applied the same experimental facilities by spatial scanning with a 1-${\rm{mm}}$ step along the *x*- and *y*-axes to characterize the fields of amplitude presented in Figs 3 and 4.

For the transmission curves in Figs 3b and 4b, we used a sweep frequency signal as a perturbation to excite the plate at the input location. The input (or output) signal can be obtained from the surface integral of the total energy flux over a rectangular cross profile containing four sites. Thus, the transmission can be given by $T\ ( \omega ) = {\rm{\ }}10{\rm{log}}( {{{\rm{W}}}_{{\rm{output}}}/{{\rm{W}}}_{{\rm{input}}}} )$ or $T\ ( \omega ) = {{\rm{W}}}_{{\rm{output}}}/{{\rm{W}}}_{{\rm{input}}} $, where ${\rm{W}}$ represents the total energy of elastic waves.

## Supplementary Material

nwac203_Supplemental_File
