# Supplementary material for: Topological materials for full-vector elastic waves
Source: Natl Sci Rev. 2022 Sep 24;10(5):nwac203. doi: 10.1093/nsr/nwac203 (PMC10124969; doi:10.1093/nsr/nwac203)
Supplement: nwac203_Supplemental_File [file nwac203_supplemental_file.docx]

**Supplementary Note for**

**Topological materials for full-vector elastic waves**

Ying Wu^1,6*^, Jiuyang Lu^1*^, Xueqin Huang^1*^, Yating Yang^1^, Li Luo^1^, Linyun Yang^2^, Feng Li^3†^, Weiyin Deng^1†^, and Zhengyou Liu^4,5†^

^1^School of Physics and Optoelectronics and State Key Laboratory of Luminescent Materials and Devices, South China University of Technology, Guangzhou, 510640, China

^2^Department of Astronautic Science and Mechanics, Harbin Institute of Technology, Harbin, Heilongjiang 150001, China

^3^Centre for quantum physics, Key laboratory of advanced optoelectronic quantum architecture and measurement (MOE), School of Physics, Beijing Institute of Technology, Beijing, 100081, China

^4^Key Laboratory of Artificial Micro- and Nanostructures of Ministry of Education and School of Physics and Technology, Wuhan University, Wuhan 430072, China

^5^Institute for Advanced Studies, Wuhan University, Wuhan 430072, China

^6^Institute of Solid Mechanics, Midea Corporate Research Center, Midea Group, Foshan, 528311, P. R. China

*Y.W., J.L. and X.H. contributed equally to this work

†Corresponding author. Email: phlifeng@bit.edu.cn; dengwy@scut.edu.cn; zyliu@whu.edu.cn

**Supplementary Note 1:** The $k\cdot p$ perturbation theory for full-vector elastic waves

**Supplementary Note 2:** Non-Abelian Wilson loop approach for full-vector elastic waves

**Supplementary Note 3:** Sample fabrication and experimental setup

**Supplementary Note 4:** Topological edge state in EMM with free boundary

**Supplementary Note 5:** Robust transport of the edge states for elastic waves

**Supplementary Note 6:** Topological interface states between A and B

**Supplementary Note 7:** Selective transport of edge states for the clamped boundary

**Supplementary Note 1: The** $\boldsymbol{k\cdot p}$ **perturbation theory for full-vector elastic waves**

For homogeneous and isotropic media, the elastic wave equation at the angular frequency of $\omega=2\pi f$ can be represented in the form of an eigenvalue equation

$$H_{0}\mathbf{u}=-c_{L}^{2}\nabla\nabla\cdot\mathbf{u}+c_{T}^{2}\nabla\times\nabla\times\mathbf{u}=\omega^{2}\mathbf{u} (1)$$

where $\mathbf{u}(\mathbf{r})$ is a vector field representing the displacement distribution and $c_{L/T}$ is the compressional/shear wave velocity. For phononic crystal, the Bloch wavevector $\mathbf{k}$ is a good quantum number, in the presence of crystalline periodicity, that can be used to index the displacement field. In general, crystalline symmetry can generate a series of degeneracies at different wavevectors in the first Brillouin zone (BZ), which is determined by the point group of the wavevector. Considering a $d$-fold degeneracy at $\mathbf{k}$ point, all the degenerated state $\mathbf{u}_{\alpha}(\mathbf{r})$ ($\alpha=1,2,\cdots,d$) constituted a basis of an $d$-dimensional representation of the point group $G_{\mathbf{k}}$. Based on the $k\cdot p$ perturbation theory, the Bloch eigenstate in the neighborhood can be represented by the degenerate ones as $\mathbf{u}_{\mathbf{k}^{\mathbf{'}}}\left( \mathbf{r} \right)\boldsymbol{=}e^{i\Delta\mathbf{k}\cdot\mathbf{r}}\sum_{\alpha=1}^{d} A_{\alpha}\mathbf{u}_{\alpha}(\mathbf{r})$ with $\Delta\mathbf{k=k'-k}$ and $A_{\alpha}$ being the expanding coefficients. Substituting $\mathbf{u}_{\mathbf{k}^{\mathbf{'}}}$ into Eq. (1), the eigenvalue equation turns out to be a perturbation form,

$$\sum_{\beta=1}^{d} H_{\alpha\beta}A_{\beta}=\sum_{\beta=1}^{d} \left[ -i\Delta\mathbf{k}\boldsymbol{\cdot}\mathbf{p}_{\alpha\beta}+\Delta\mathbf{k}\Delta\mathbf{k}\mathbf{:}q_{\alpha\beta} \right]A_{\beta}=2\omega_{\mathbf{k}}\Delta\omega A_{\alpha}, (2)$$

in which the vector term $p_{i,\alpha\beta}=\int\left[ {(c}_{L}^{2}-c_{T}^{2})(u_{i,\alpha}^{*}\partial_{k}u_{k,\beta}+u_{k,\alpha}^{*}\partial_{k}u_{i,\beta})+2c_{T}^{2}u_{k,\alpha}^{*}\partial_{i}u_{k,\beta} \right]dV$, the tensor term $q_{ij,\alpha\beta}=\int\left[ {(c}_{L}^{2}-c_{T}^{2})u_{i,\alpha}^{*}u_{j,\beta}+c_{T}^{2}{\delta_{ij}u}_{k,\alpha}^{*}u_{k,\beta} \right]dV$

, and $\Delta\omega=\omega_{\mathbf{k'}}-\omega_{\mathbf{k}}$. Here Einstein summation convention is used and $u_{i,\alpha}$ means the $i$th component of the $\alpha$th function in the representation basis. Constrained by symmetry, the components of $\mathbf{p}$ and $q$ are not all independent. For $\mathbf{p}$, it has been proven [1] that

$$p_{i,\alpha\beta}=p_{j,\alpha^{'}\beta^{'}}R_{ji}D_{\alpha^{'}\alpha}^{*}\left( R \right)D_{\beta^{'}\beta}\left( R \right), (3)$$

where the matrix $R$ is an element of $G_{\mathbf{k}}$ that represents a point operation (e.g., rotation and inversion) and $D(R)$ is the associated representation matrix of $R$. Employing a similar procedure, we obtain the symmetry constraints for $q$ as

$$q_{ij,\alpha\beta}=q_{mn,\alpha^{'}\beta^{'}}R_{im}R_{jn}D_{\alpha^{'}\alpha}^{*}\left( R \right)D_{\beta^{'}\beta}\left( R \right). (4)$$

Eq. (3) and (4) establish respectively the relations among the different components of $\mathbf{p}$ and $q$, which fully simplifies the perturbation problem in Eq. (2).

For one single layer of our phononic crystal, we focus on the M point in the first BZ, whose point group is $C_{4v}$. This point group has eight elements, including the identity, the inversion, the clockwise and anticlockwise rotations of rotations by $\pi/2$, and two pairs of mirror operations (vertical and diagonal). The corresponding matrix $R$ of each point operator can be straightforwardly obtained, which mathematically equals the 2D irreducible representation $D(R)$ when the basis functions are chosen as $x$ and $y$. Substituting $R$ and $D(R)$ into Eq. (3), we obtain zeros for all the components of $\mathbf{p}$, which means no conic dispersions can survive in the vicinity of the M point and implies the dispersions there are quadratic depending on $q$. Further using Eq. (4), part of the components of $q$ are restricted to zero, while the nonzero terms are not independent with the relationships as $q_{xx,11}=q_{yy,22}$, $q_{yy,11}=q_{xx,22}$, $q_{yx,12}=q_{xy,21}$, and $q_{xy,12}=q_{yx,21}$. Introducing the parameters $q_{0}=(q_{xx,11}+q_{yy,11})/2$, $q_{1}=(q_{xy,12}+q_{yx,12})/2$ and $q_{2}=(q_{xx,11}-q_{yy,11})/2$, the perturbation Hamiltonian for one single layer can be finally reduced into

$$\delta H^{'}=\left( \Delta k_{x}^{2}+\Delta k_{y}^{2} \right)q_{0}+2\Delta k_{x}\Delta k_{y}q_{1}\sigma_{x}+\left( \Delta k_{x}^{2}-\Delta k_{y}^{2} \right)q_{2}\sigma_{z}, (5)$$

where $\sigma_{i}$ are Pauli matrices and ${(\Delta k}_{x},{\Delta k}_{y})$ is the dimensionless wavevector deviating from the M point.

Without interlayer couplings, the Hamiltonian of bilayer phononic crystal, $\delta H$, is simply a direct sum of two single layer ones, i.e., $\delta H=\delta H^{'}\bigoplus\delta H^{'}$. With the chiral interlayer couplings depicted in Fig. 1c, the dominate couplings stem from the $x$ (or $y$) polarized mode in the upper layer with the $y$ (or $x$) polarized mode in the lower layer, which contributing to $\eta$ (or $-\eta$) to anti-diagonal terms of $\delta H$. A more specific description involves the couplings between $x$ (or $y$) polarized mode in the upper layer with the $x$ (or $y$) polarized mode in the lower layer, which slightly lifts the dispersion degeneracies and is negligible to topological analysis. Therefore, the Hamiltonian of bilayer phononic crystal including both intralayer and interlayer couplings reads,

$$\delta H=\left( \Delta k_{x}^{2}+\Delta k_{y}^{2} \right)q_{0}+2\Delta k_{x}\Delta k_{y}q_{1}\sigma_{x}+\left( \Delta k_{x}^{2}-\Delta k_{y}^{2} \right)q_{2}\sigma_{z}+\eta\tau_{y}\sigma_{y}, (6)$$

where Pauli matrices $\tau_{i}$ are further introduced to indicate the layer pseudospins.

**Supplementary Note 2: Non-Abelian Wilson loop approach for full-vector elastic waves**

The Eigen elastic waves propagating in our bilayer metamaterial is of the Bloch wave form, i.e., $\mathbf{u}_{n,\mathbf{k}}={\hat{\mathbf{u}}}_{n,\mathbf{k}}\exp\left( i\mathbf{k}\cdot\mathbf{r} \right)$, here $\mathbf{u}_{n,\mathbf{k}}$ is the Bloch elastic wave function of the actual displacement fields, and ${\hat{\mathbf{u}}}_{n,\mathbf{k}}$ represents the corresponding cell periodic counterpart obtained by COMSOL Multiphysics. With the inner products between vectors at different $\mathbf{k}$ defined as $\left\langle{\hat{\mathbf{u}}}_{n,\mathbf{k}}|{\hat{\mathbf{u}}}_{n,\mathbf{k}^{\boldsymbol{'}}} \right\rangle=\int_{\mathrm{cell}} \rho{\hat{\mathbf{u}}}_{n,\mathbf{k}}^{*}\cdot{\hat{\mathbf{u}}}_{n,\mathbf{k}^{\boldsymbol{'}}}d\mathbf{r}$, the Berry connection and the Berry curvature are given by $\mathbf{A}_{n}=\left\langle{\hat{\mathbf{u}}}_{n,\mathbf{k}}|i\nabla_{k}|{\hat{\mathbf{u}}}_{n,\mathbf{k}^{\boldsymbol{'}}} \right\rangle$ and $\Omega_{n}\left( k \right)=\partial_{k_{x}}A_{k_{y}}-\partial_{k_{y}}A_{k_{x}}$, respectively. Then we can calculate the Berry phase by integrating the Berry curvature over the entire Brillouin zone,

$$\Phi_{n}=\int_{\mathrm{BZ}} \Omega_{n}\left( k \right)d\mathbf{k}={\phi_{n}^{{(k}_{2})}(k_{1})|}_{0}^{2\pi/a}, (7)$$

where $\phi_{n}^{\left( k_{2} \right)}\left( k_{1} \right)=\int_{0}^{2\pi/a} A_{k_{2}}{dk}_{2}$ is a function of $k_{1}$which runs within a closed loop from $0$ to $2\pi/a$. Here $(k_{1},k_{2})$ is a proper choice of $(k_{x},k_{y})$ such that the integration of $\mathbf{A}_{n}$ along direction $k_{2}$ is well defined. We would like to note that the periodic property in $\mathbf{k}$-space of the Berry connection has been applied to reach the final result in Eq. (7). Since it is at the same physical state for $k_{1}$ equal to $0$ and $2\pi/a$, we can expect that phase angle $\phi_{n}^{\left( k_{2} \right)}$ must be identical at the ending points, up to a phase accumulation of $2\pi m$ for some integer $m$. Based on this scenario, we are informed by an important fact that Chern number is just identical to the integer $m$, which is the Berry phase divided by $2\pi$.

Since the solutions of wave equation are generally performed on a discrete mesh of $\mathbf{k}$ points sampled within the Brillouin zone, it is more useful to consider the discretized expression of $\phi_{n}^{\left( k_{2} \right)}$,

$$\phi_{n}^{\left( k_{2} \right)}(k_{1})=-Im ln\prod_{k_{2}=0}^{2\pi/a} \left\langle\mathbf{u}_{n,k_{2}}|\mathbf{u}_{n,k_{2}+\Delta k_{2}} \right\rangle, (8)$$

in which the inner product between two neighboring $\mathbf{k}$ points is required to construct the phase $\phi_{n}^{\left( k_{2} \right)}$. Then we can trace the evolution of $\phi_{n}^{\left( k_{2} \right)}$ when $k_{1}$ runs from $0$ to $2\pi/a$ and obtain the Berry phase according to Eq. (8).

The description above is only valid for an isolated band, which means the *n*th band does not touch with the $(n-1)\mathrm{th}$ or $(n+1)\mathrm{th}$ bands at any point throughout the entire Brillouin zone. But degeneracies do occur in our case, and at those points, hybridization of the degenerate modes obscures the application of Eq. (8). In such cases, instead of considering one single band, we have to take both the two degenerated bands into account together as a group. Therefore, the inner product in Eq. (8) is no longer a scalar, but a 2-by-2 matrix with its entries be $\left\langle\mathbf{u}_{m,k_{2}}|\mathbf{u}_{n,k_{2}+\Delta k_{2}} \right\rangle$, in which band indices $m$, $n$ run in the considered 2-band group. And the Berry phase should be understood as a total Berry phase,

$$\phi_{\mathrm{tot}}^{\left( k_{2} \right)}(k_{1})=-Im ln det\prod_{k_{2}=0}^{2\pi/a} M_{{k_{2}, k}_{2}+\Delta k_{2}}, (9)$$

One can also extract the individual Berry phase by taking the eigenvalue the resultant overlap matrix $M$. It is obvious that $\phi_{\mathrm{tot}}=\sum\phi_{n}$. The above approach is referred to non-Abelian Wilson loop method for elastic wave [2]. To corroborate the topological property of the system, we extract the Bloch wave functions of the first two flexural bands below the gap ($<25.4 kHz$), namely, the first red curves in the band structure. Supplementary Figure 1 provides the evolution of the Wannier function centers (Berry phases $\theta$) with respect to wavevector $k_{x}$ and distinctly shows the gapless property in the Brillouin zone. The Wannier bands enclosing the whole $k_{x}-\theta$ cylinder one time indicates that the bandgap is of topological non-trivial $Z_{2}$ index [3].


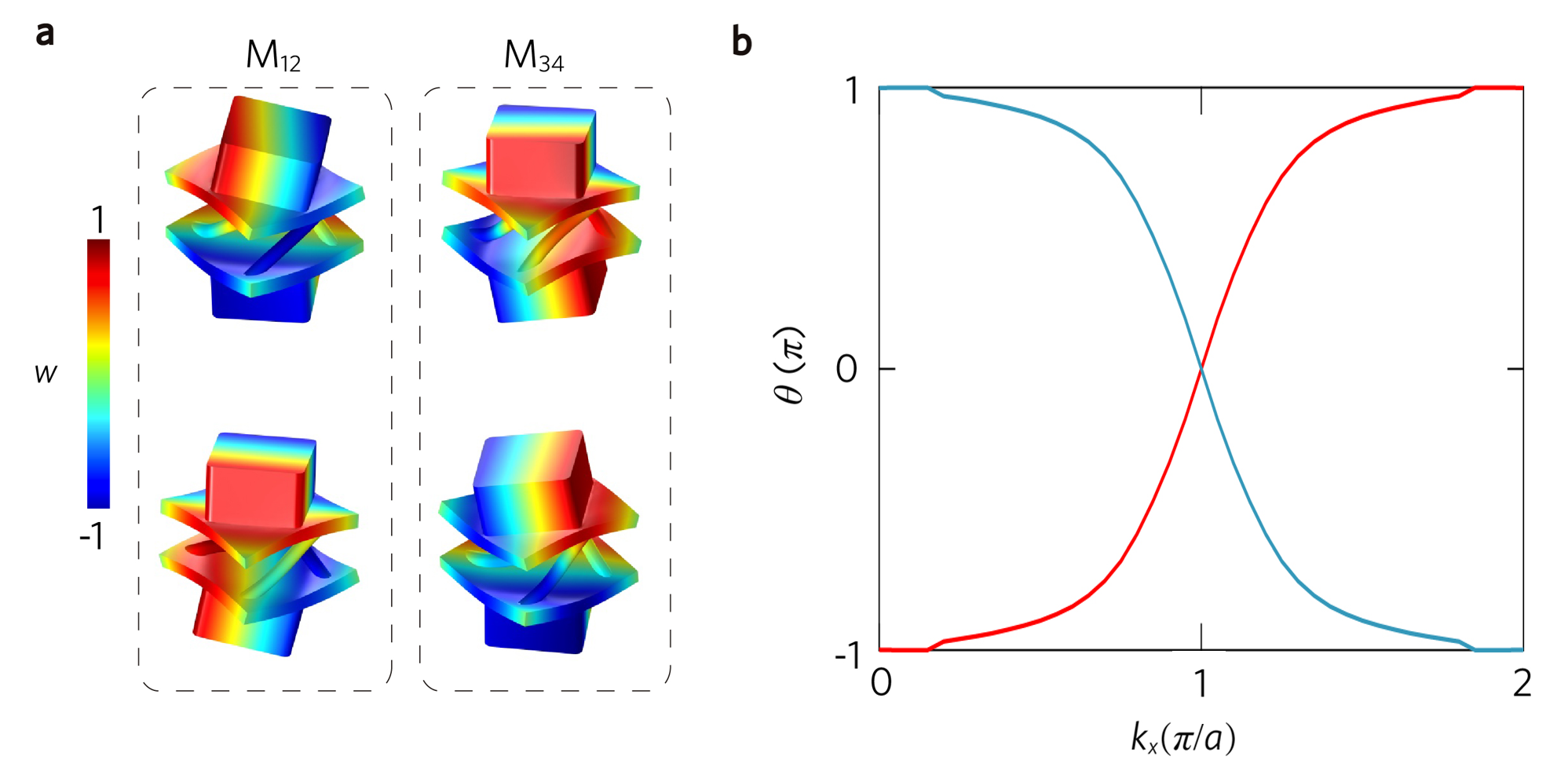


**Supplementary Figure 1: Eigenmodes and** **Non-Abelian Wilson loop. a**, Eigenmodes at the $M$ point. The color map represents the out-of-plane deformation. **b**, The evolution of the Wannier centers with respect to $k_{x}$ for the first two bands, corresponding to the inset of Fig. 1d in the main text.

**Supplementary Note 3: Sample fabrication and experimental setup**

The sample is fabricated by the 3D metal printing technology, specifically the selective laser melting technology [4]. Supplementary Figure 2a shows the schematic diagram of the technology. Our sample is fabricated layer by layer with melting metal powder. It is worth noting that we have to place the sample at a 45-degree angle to the building platform (see Supplementary Figure 2b) in order to avoid support structures staying in the seam. After removing the outside support structures carefully, we can obtain the desired sample, as shown in Supplementary Figure 2c.

Supplementary Figure 3a shows the experimental setup. The displacement field is accurately measured by a laser vibrometer (Polytec OFV-5000), which is mounted on a 2D automatic scanning stage with a step of 1 mm. During scanning the laser beam from the vibrometer is perpendicular to the sample and only the vertical displacement component (z-component) is captured. The displacement sensitivity of the decoder is 50 nm/V. The displacement signal from the vibrometer is further accurately recorded by a network analyzer (Keysight E5061B with output resistance 50). For vibration excitation, we attach a piezoelectric ceramic disc to the sample as a point source. All the instruments are connected with the computer for real-time monitoring, automatic scanning, and signal processing. Here is an example, the original measurements of flexible transport with $H=14a$, shown in Supplementary Figure 3b. The anomalous bright spots presented in the result are noise disturbance, which has been removed by the 2D Gaussian filtering method.


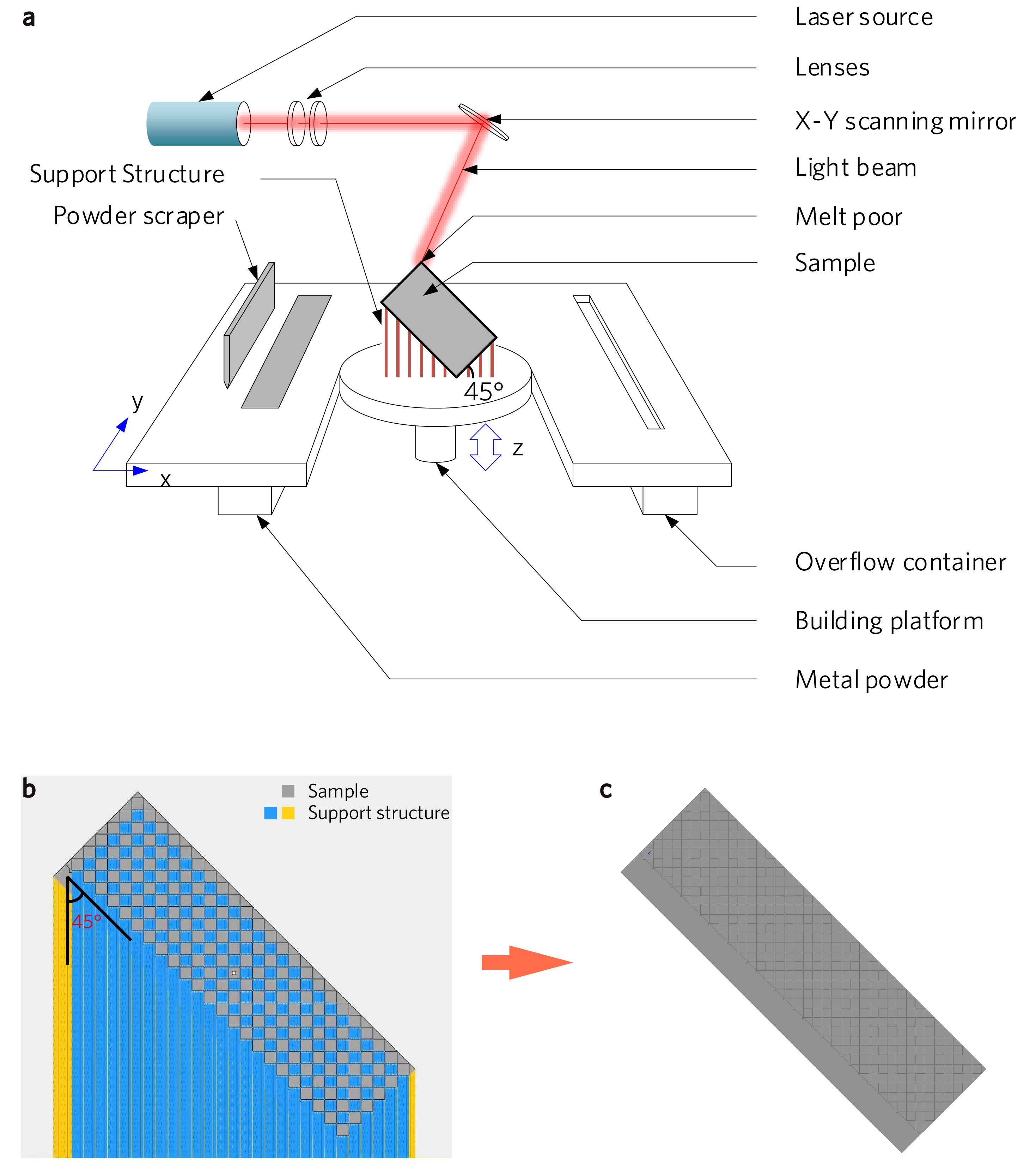


**Supplementary Figure 2: Sample fabrication.** **a**, A schematic diagram of 3D metal printing based on the selective laser melting technology. **b**, The sample is placed at a 45 degree angle to the building platform. The blue and yellow regions denote the support structures, which will be removed after finishing printing. **c**, Sample after removal of support.


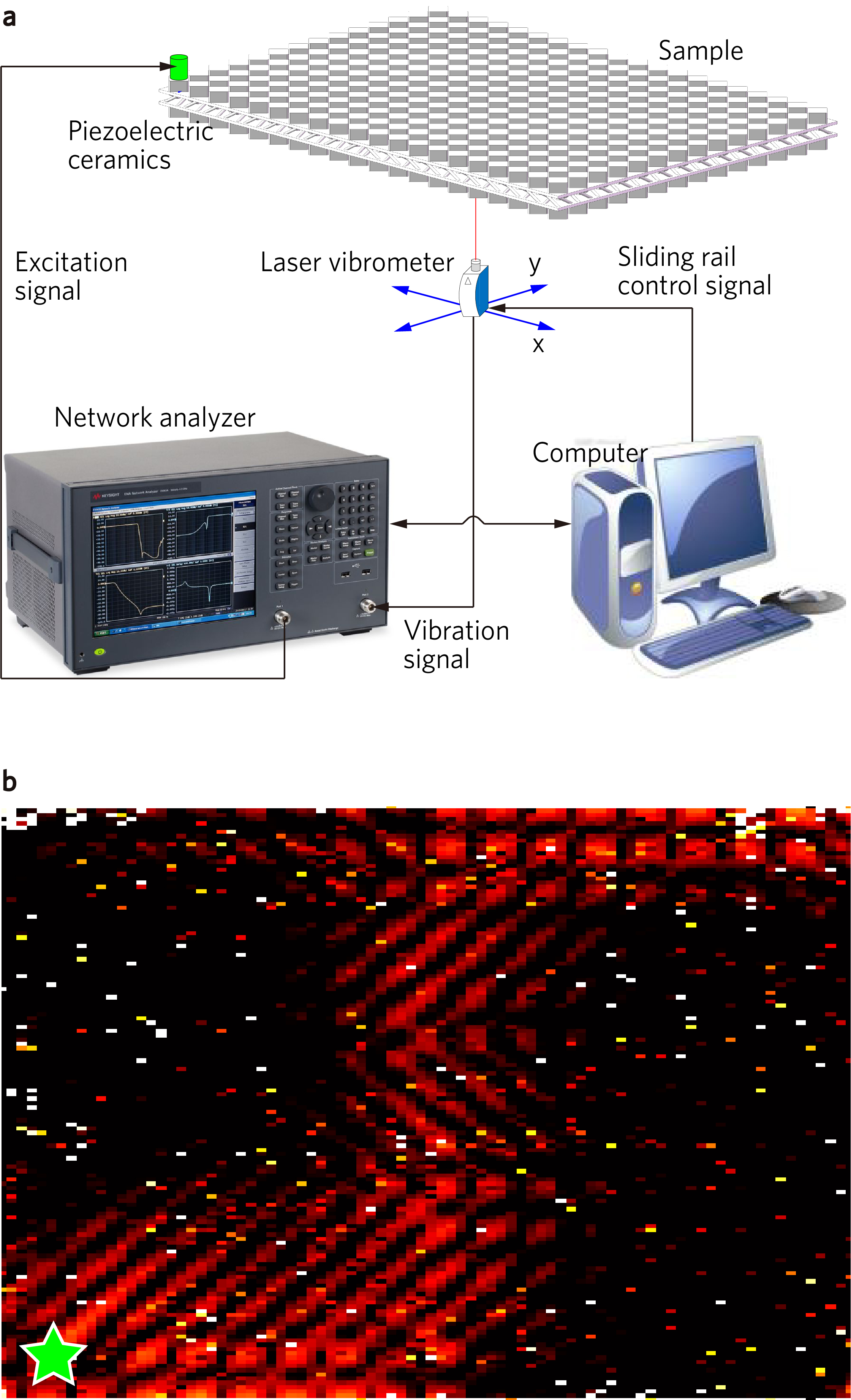


**Supplementary Figure 3: Experiment setup and measurement.** **a**, Schematics of experimental facilities. **b**, The scanned displacement field distribution at $26.75 kHz$. The green star denotes the source.

**Supplementary Note 4: Topological edge states in EMM with free boundary**

In the main text, we find that there is a tiny gap appearing in the edge state dispersions on the free boundary. In this regard, the backscattering is not completely forbidden in the experiment. However, the real issue is whether the experimental measurement can detect such a tiny bandgap. We observed that in our work is that the transmission through the topological waveguide is nearly unaffected (Fig. 3 in the main text), suggesting that the bandgap is hardly noticeable and the backscattering is largely suppressed. Here, we present additional simulated results of the elastic edge states propagating along mixed boundaries, including both free and clamped boundaries. Supplementary Figure 4 shows field distributions. We can find that even though the exciting frequency is set just within the gap ($27.62 kHz\sim27.72 kHz$), the elastic helical wave can also propagate smoothly along the sharp corners both on the free and clamped boundaries. Therefore, the tiny gap is yet too small and cannot affect the robust transmission in the experiments or simulations.

Interestingly, the gapless helical states can also exist at the $45^{\circ}$ boundary, which further proves the robustness of our system. The projected dispersions and the eigenmodes of the zigzag supercell are illustrated in Supplementary Figure 5.


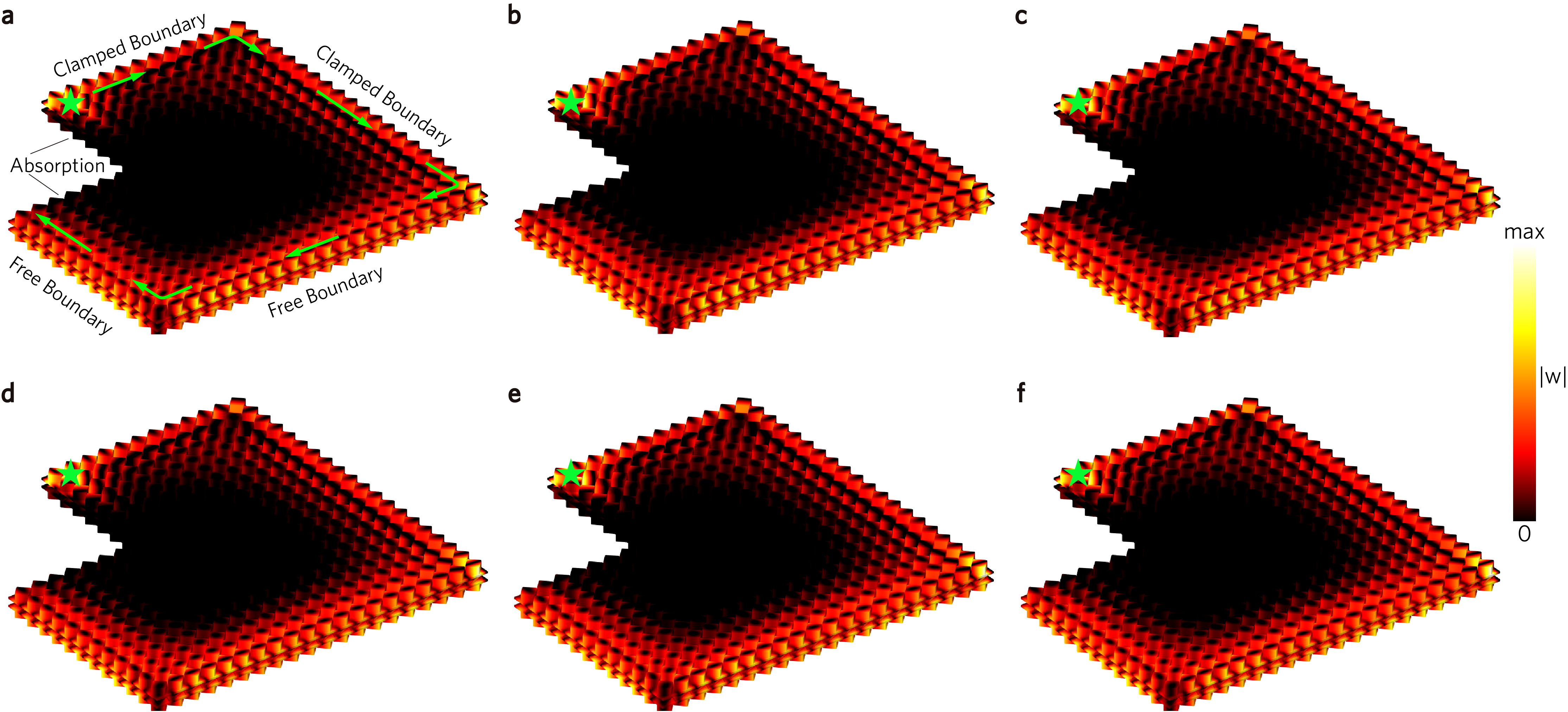


**Supplementary Figure 4: Topological protected edge states at frequencies inside the tiny gap appeared in the projected dispersions on the free boundary. a**, There are three kinds of boundaries, namely clamped, free, and absorption. The source is marked by a green star with frequency $f=27.62 kHz$. **b**, $f=27.64 kHz$. **c**, $f=27.66 \mathrm{kHz}$. **d**, $f=27.68 \mathrm{kHz}$. **e**, $f=27.70 kHz$. **f**, $f=27.72 kHz$.


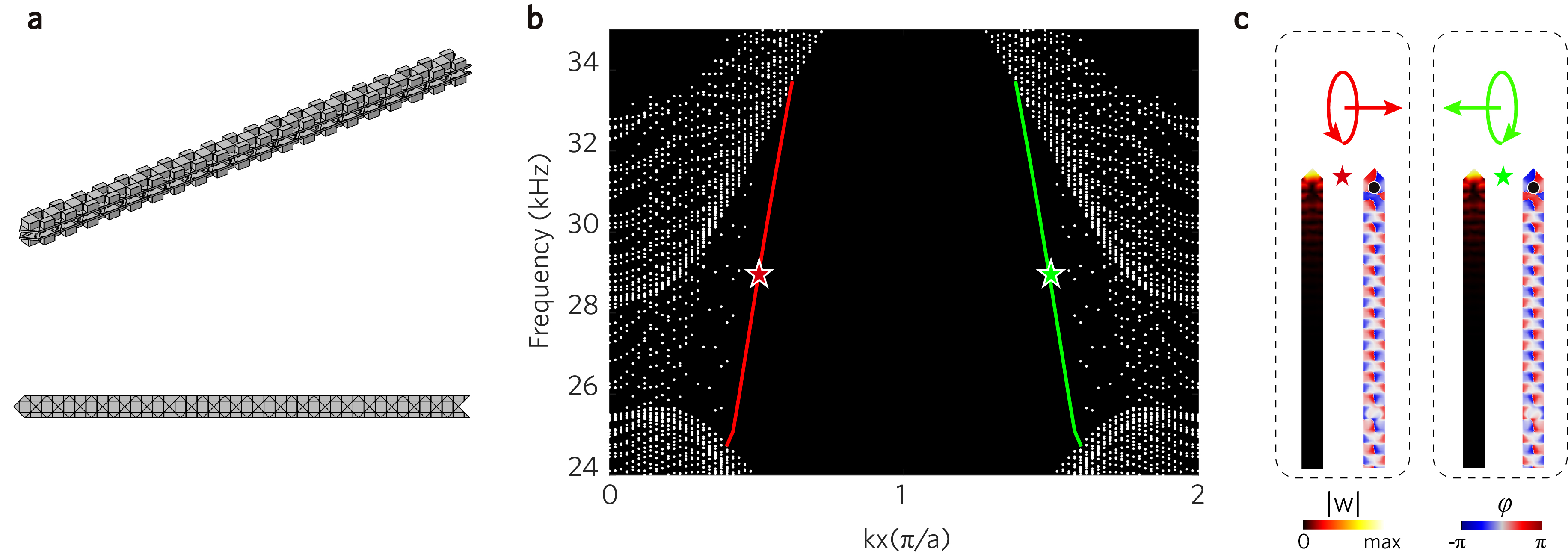


**Supplementary Figure 5: Topological edge states along the** $\boldsymbol{45^{\circ}}$ **boundary.** **a**, Supercell. Floquet-Bloch periodic condition is applied with lattice constant equal to $\sqrt{2}a$. **b**, Projected dispersions of edge states. The solid red and green lines denote a pair of edge states counter-propagating along the boundary. **c**, Out-of-plane amplitude ($\left| w \right|$) and phase ($\varphi$) field of the helical edge states marked by two stars in **b**. Likewise, there also exists vortices in the phase profiles.

**Supplementary Note 5: Robust transport of the edge states for elastic waves**

In the main text, we have demonstrated the reflection immunity of elastic edge waves against a rectangular defect for the free boundary. Here, we present the simulation result for the clamped boundary. We excite chirped signals with frequency ranging from $23.5 \mathrm{kHz}$ to $30.5 \mathrm{kHz}$. Supplementary Figures 6a and 6b show the transmissions of the defect path with ideal clamped boundaries. The high transmission of the elastic edge waves within the topological gap (shadow region) demonstrating weak backscattering along the rectangular defect.

In mechanical systems, a structure is usually clamped by much harder material to achieve large acoustic impedance mismatch and realize the fixed boundary condition. In the paper, for the experimental realization of the clamped boundary, we designed an extension part to be fixed. However, since the extension part and the bulk structure share the same material, the large impedance mismatch cannot be ideally satisfied. Supplementary Figure 6c shows the elastic boundary wave propagates along the sharp corners along the rectangle defect with the extended part clamped. The intensity of wave energy on the right side is lower than the left side, which implied that some energy will leak into the extension part, which inevitably gives rise to backscattering. Therefore, it is not suitable to experimentally verify the backscattering immunity of elastic waves on the clamped boundaries.

On the other side, the zoom-in region of the straight path indicates that there is little energy flow into the well-clamped extension parts. We can employ a straight well-clamped sample to approximate the ideal fixed boundary and carry out the topological edge states in the experiment. The efficiency and reliability of such clamped condition are further proved by the agreement between the simulated and experimental dispersion curves in Fig. 2e in the main text.

In addition, we utilize multiple sources with specific phases to realize selectively excitation of the pseudospin up and down edge states, as shown in Supplementary Figure 7. For the clamped boundary, we apply three sources with phases equal to $-\pi/3$, $0$, $\pi/3$. Besides, four sources ($\varphi={3\pi}/2$, $\varphi=\pi$, $\varphi=\pi/2$, $\varphi=0$) are used to excite the unidirectional transport on free boundary. The sequences of the phases need to be reversed in order to excite the opposite directions. The simulated results indicate the intriguing phenomenon, one-way transport, can also be reproduced in our EMM by strategic excitations.

It should be noted that the elastic waves in our system, which is not a thin-plate structure, cannot decouple into the in-plane and out-of-plane modes, especially in the presence of the heavy square blocks and non-negligible tilted pillars. Actually, the full-vector property is essential for the nontrivial topology. It embodies the form of the effective Hamiltonian and has been applied in non-Abelian Wilson-loop calculation.

On the other hand, we perform a simulation to show that the robust edge states can also be stimulated by an in-plane excitation. As shown in Supplementary Figure 8, the excitation is applied by a point source with only the in-plane polarization along the $x$ direction. The edge state, containing both in-plane and out-of-plane components, can propagate smoothly against sharp corners, manifesting its robustness feature.


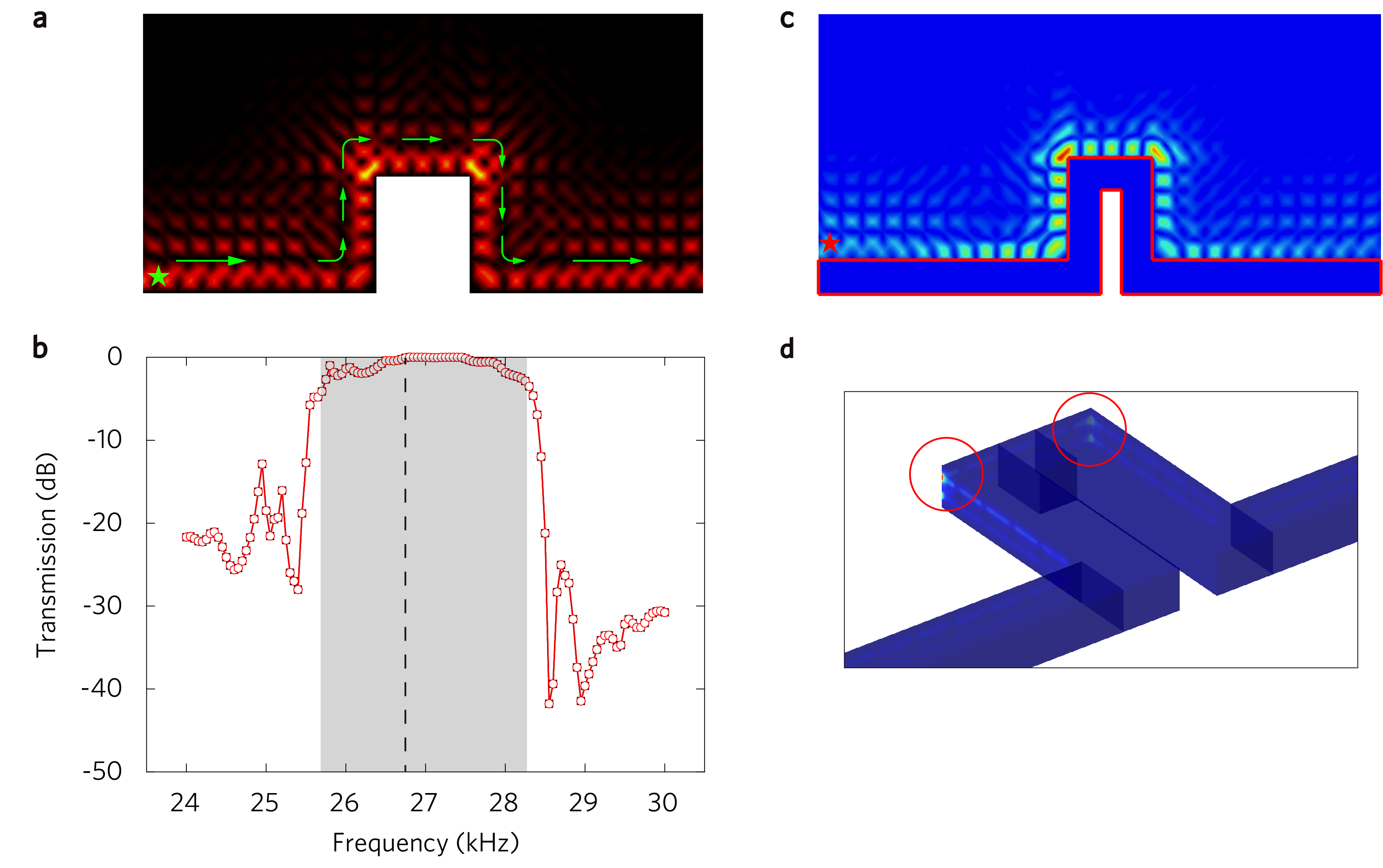


**Supplementary Figure 6: Robust transport of elastic wave along clamped boundaries with a rectangle defect.** **a**, Excitation frequency $f=26.75 kHz$. The green star denotes the source position. **b**, Calculated transmission of the path with the defect. For simulation, since there is no loss factor and the boundary can be clamped ideally, the transmission rate is almost equal to 1 indicating that negligibly weak backscattering of elastic edge states. **c**, Simulation of the experimentally realized clamped boundary with rectangle defect, the distribution of elastic wave is not even. The zoom-in regions for the corners are shown in **d**.


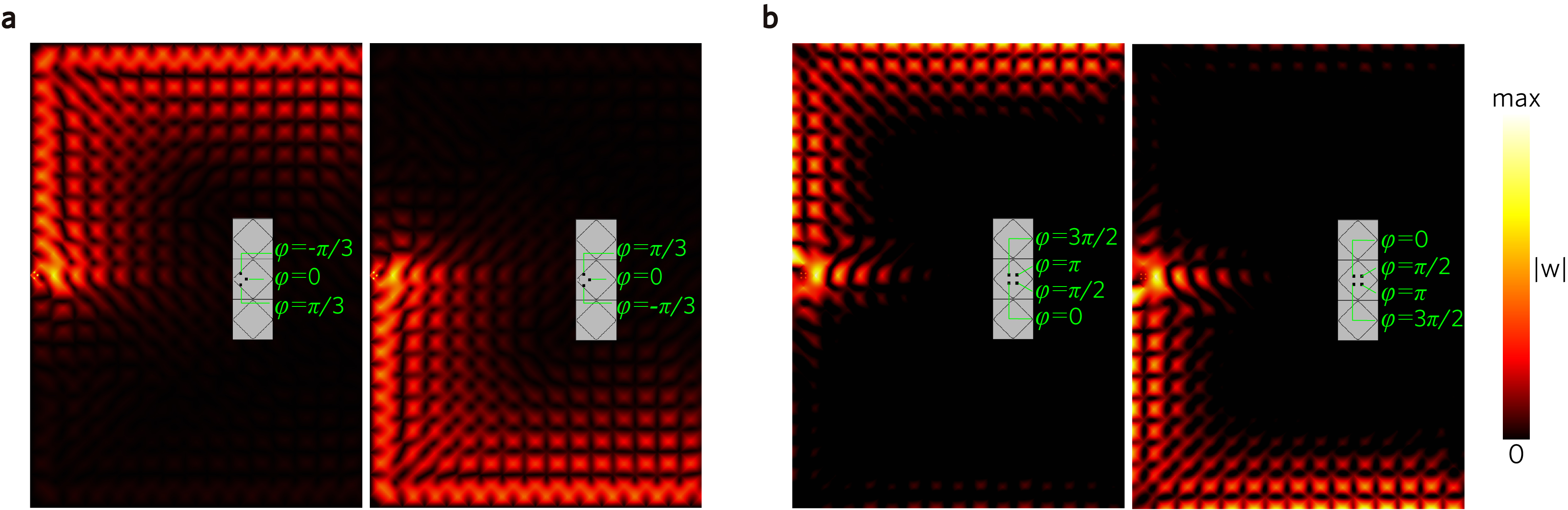


**Supplementary Figure 7: Unidirectional propagation of topologically protected helical edge states. a**, Clamped boundary. **b**, Free boundary. Multiple point sources with phase differences are applied to selectively excite the helical edge states.


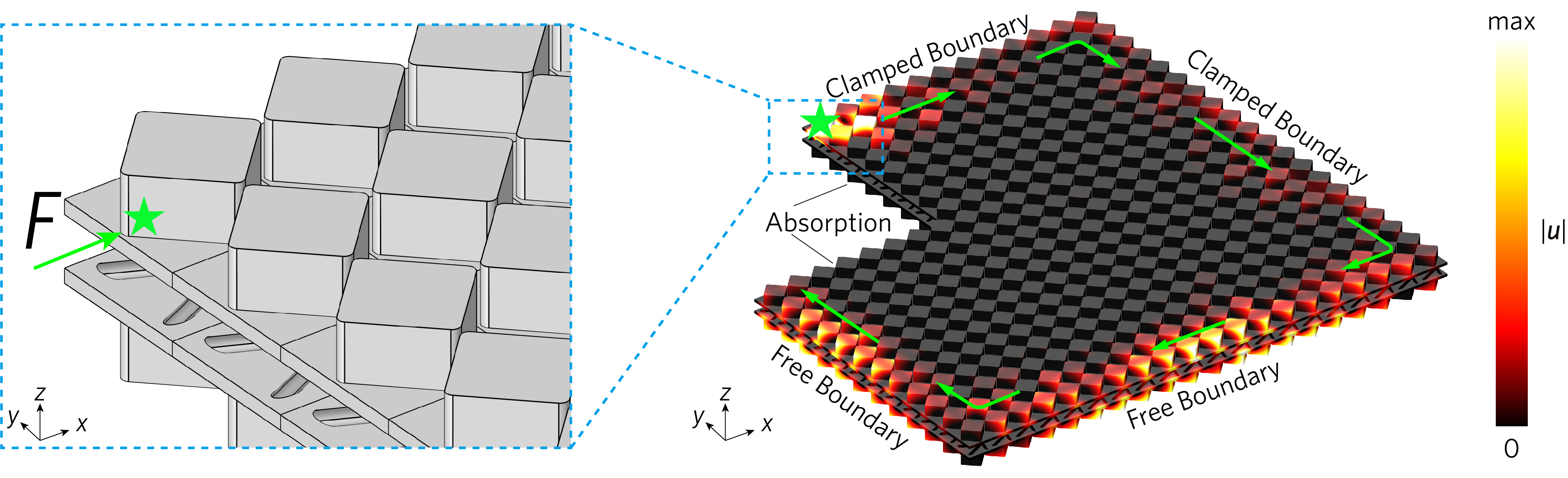


**Supplementary Figure 8:** Topological protected edge states by in-plane excitation at frequency of 26.75 kHz. Left panel: the zoom-in in-plane force excitation along the $x$ direction. Right panel: the field of the total displacement $\left| \boldsymbol{u} \right|$. The green star denotes the source. The green arrows show the direction of wave propagation.

**Supplementary Note 6: Topological interface states between A and B**

In the main text, we have shown the topological properties of A experimentally and theoretically. Here, we will present a further discussion about the topological interface states between A and B. As shown in Supplementary Figure 9, A and B share the same characters and symmetry, resulting in the same band structures. But their topological properties are reverse, which is manifested by the fact that the two pairs of degenerate vibration modes presented at $M$ point are inverse. The reversed topology gives rise to the existence of four topological interface states between A and B, further confirming the topological nature of our elastic topological metamaterial.


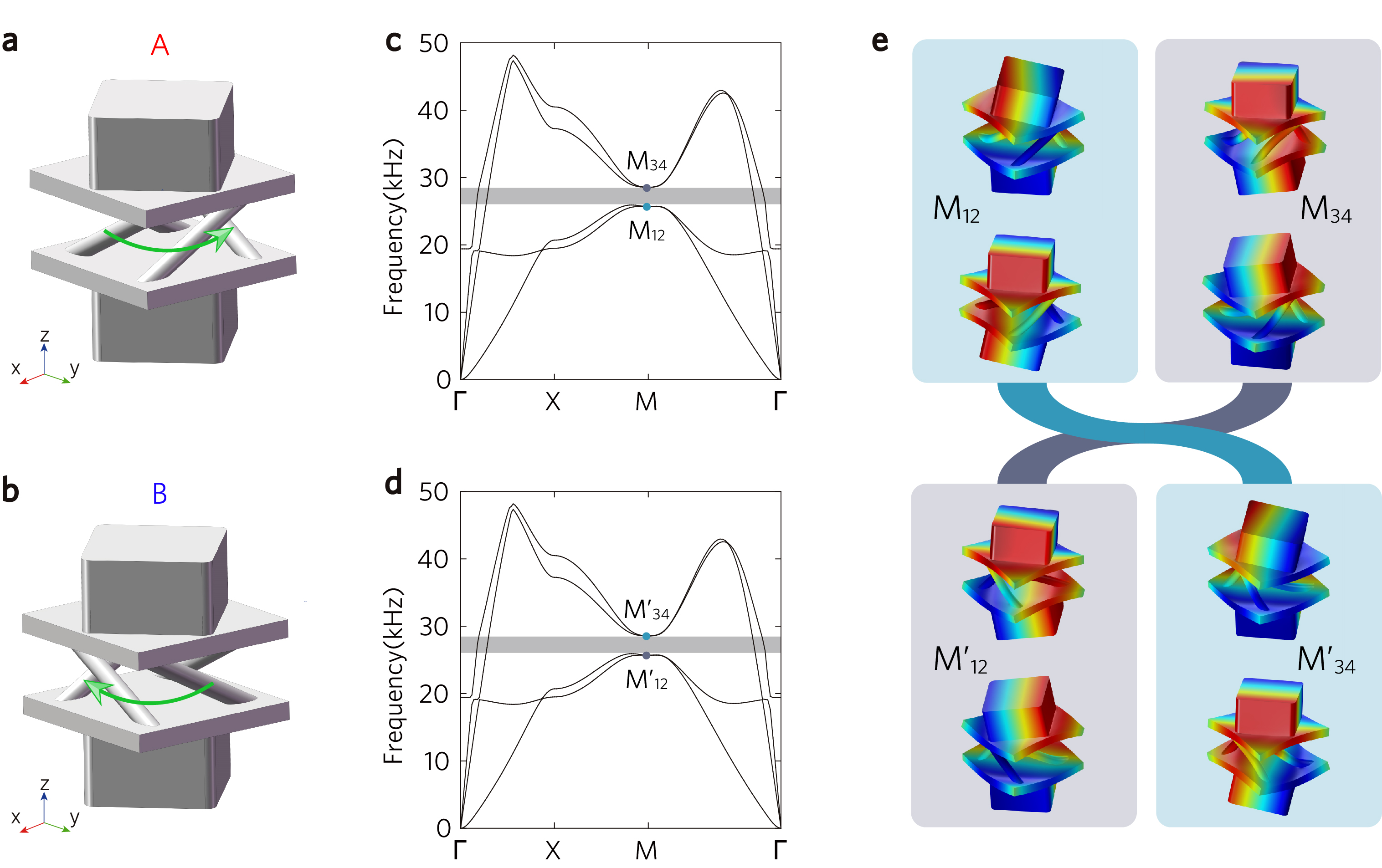


**Supplementary Figure 9: Comparison between A and B structures.** **a** and **b**, Unit cells with clockwise and anticlockwise chiral interlayer couplings, respectively. **c** and **d**, The band structure of A and B. **e**, The eigenstates at M points marked by $M_{12}$ and ${M'}_{34}$ or $M_{34}$ and ${M'}_{12}$, are flipped.

We construct a sample to form an interface and measure the amplitude and phase to experimentally obtain the edge spectrum. As observed in Supplementary Figure 10a, four topological interface states emerge at the interfaces. The calculated dispersions and experimental results match well with each other, verifying the presence of topological states, and further confirming the topological nature of our elastic topological metamaterial. The solid green and red lines denote two pairs of interface waves counter-propagating along the interface. The broken $C_{4}$ symmetry at the interface causes the coupling and mixing of pseudospin up and down interface states, inducing a small bandgap in the boundary state dispersions [5]. In reality, any fabrication imperfection could lead to a small gap. Such a gap can even be closed with a deliberately designed linking layer, as shown in Supplementary Figure 10b. The length of the linking layers is $\delta=0.2a$. With such a small change, which modifies the interface potential, the gapped interface states turn into gapless ones. By utilizing the coupling effects between these topological interface states, elastic wave manipulation and selective transport can be eventually achieved.

These topological interface states can give rise to a topological device, as discussed in Fig. 4 in the main text, in which the transmission of output ports 3 and 4 implies wane and wax as the height changes. To explain the oscillation phenomenon, as shown in Supplementary Figure 9a, we fix a frequency in the band gap and assume two forward edge states at the interface as $\psi_{1}$ and $\psi_{2}$, whose periodic parts are denoted as $u_{1}$ and $u_{2}$, and wave vectors are $k_{1}$ and $k_{2}$ ($k_{2}=k_{1}+\Delta k$, $\Delta k\approx0.24\pi/a$) along the $y$ direction. In this basis, a general interface state $\psi$ propagating along the $x$ direction can be expressed in a form of $\psi=c_{1}u_{1}e^{ik_{1}y}+c_{2}u_{2}e^{ik_{2}y}$, where $c_{1}$ and $c_{2}$ are constants, determined by excitation. Because of the mirror symmetry along the $y$ direction, the two states $u_{1}$ and $u_{2}$ have definite parity: even and odd respectively. Thus, these two states are no longer belong mainly to A or B in the presence of coupling, but can be decomposed as $u_{1}=\left( u_{+}+u_{-} \right)/\sqrt{2}$ and $u_{2}=\left( u_{+}-u_{-} \right)/\sqrt{2}$, where $u_{+}$ and $u_{-}$ are another two states weighting at $x>0$ and $x<0$, respectively. For the case shown in Fig. 4 in the main text, the excitation is exerted at $x<0$ on the bottom end, that is, $\psi\left( y=0 \right)=e^{i\phi}u_{-}$ ($\phi$ is an arbitrary phase for excitation), we have $c_{1}=-c_{2}={e^{i\phi}}/\sqrt{2}$. Therefore, $\left| \left\langle u_{-} | \psi\right\rangle\right|^{2}=\cos^{2} [(\Delta ky-\phi)/2]$ and $\left| \left\langle u_{+} | \psi\right\rangle\right|^{2}=\sin^{2} [(\Delta ky-\phi)/2]$. This means that on the $x<0$ side, the excited state oscillates as $\cos^{2} [(\Delta ky-\phi)/2]$, while on the $x>0$ side, it oscillates as $\sin^{2} [(\Delta ky-\phi)/2]$. Such oscillated state finally transports to output ports 3 and 4 with the strength at the top end, i.e., $\cos^{2} [(\Delta kH-\phi)/2]$ for port 3 and $\sin^{2} [(\Delta kH-\phi)/2]$ for port 4. The period of wave energy is a period ${2\pi}/{\Delta k}\approx8a$, which explains the oscillation period of transmission at ports 3 and 4. Fitting the simulation data in Fig. 4b in the main text, we can find that $\phi\approx{4\pi}/3$.


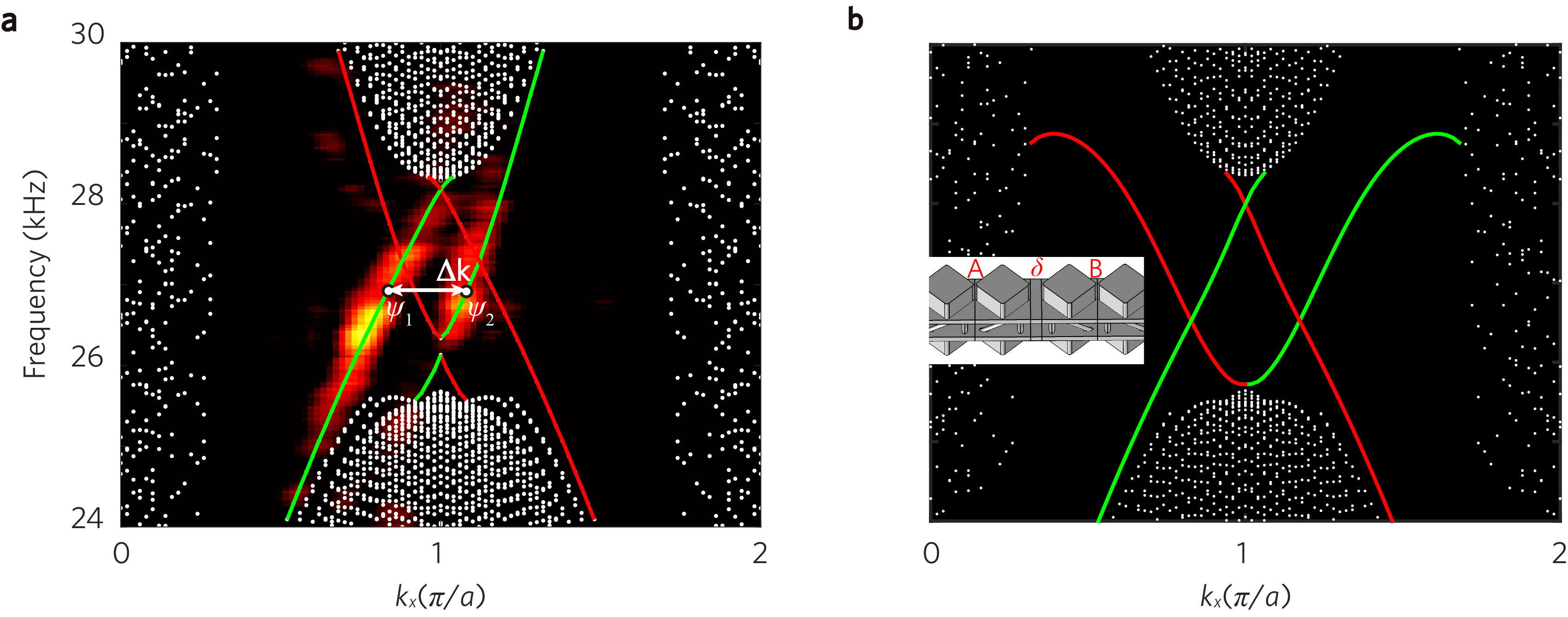


**Supplementary Figure 10**: **Topological interface states between A and B.** **a**, Projected dispersions of a supercell composed of A and B. $\Delta k$ denotes the difference of wavevectors between two interface states (denoted by the two black cycles) in the same direction. **b**, Projected dispersions of a supercell composed of A, B, and linked by two small pieces of pure plates.

**Supplementary Note 7: Selective transport of edge states for the clamped boundary**

Here, we present the simulated results of the flexible transport of elastic waves for the clamped boundary. Supplementary Figure 11a shows the schematic diagram and the calculated energy flow of port 2-4 versus height. Different from the free boundary, the top and bottom sides of the sample are clamped. Although the transmission curves of the two boundary conditions share the same periods, i.e., $8a$, their shapes are shifting. For example, when $H=16a$, for the free boundary, the wave energy is splitting into port 3 and 4. While for the clamped boundary, almost all the elastic wave propagates into the port 3. As shown in Supplementary Figure 11b-d, we present the simulation results of the out-of-plane displacement for three typical heights, namely $H=16a$, $18a$, and $20a$, respectively. Our calculation further indicates an intriguing phenomenon that the transmission of ports 3 and 4 implies wane and wax as the height changes.


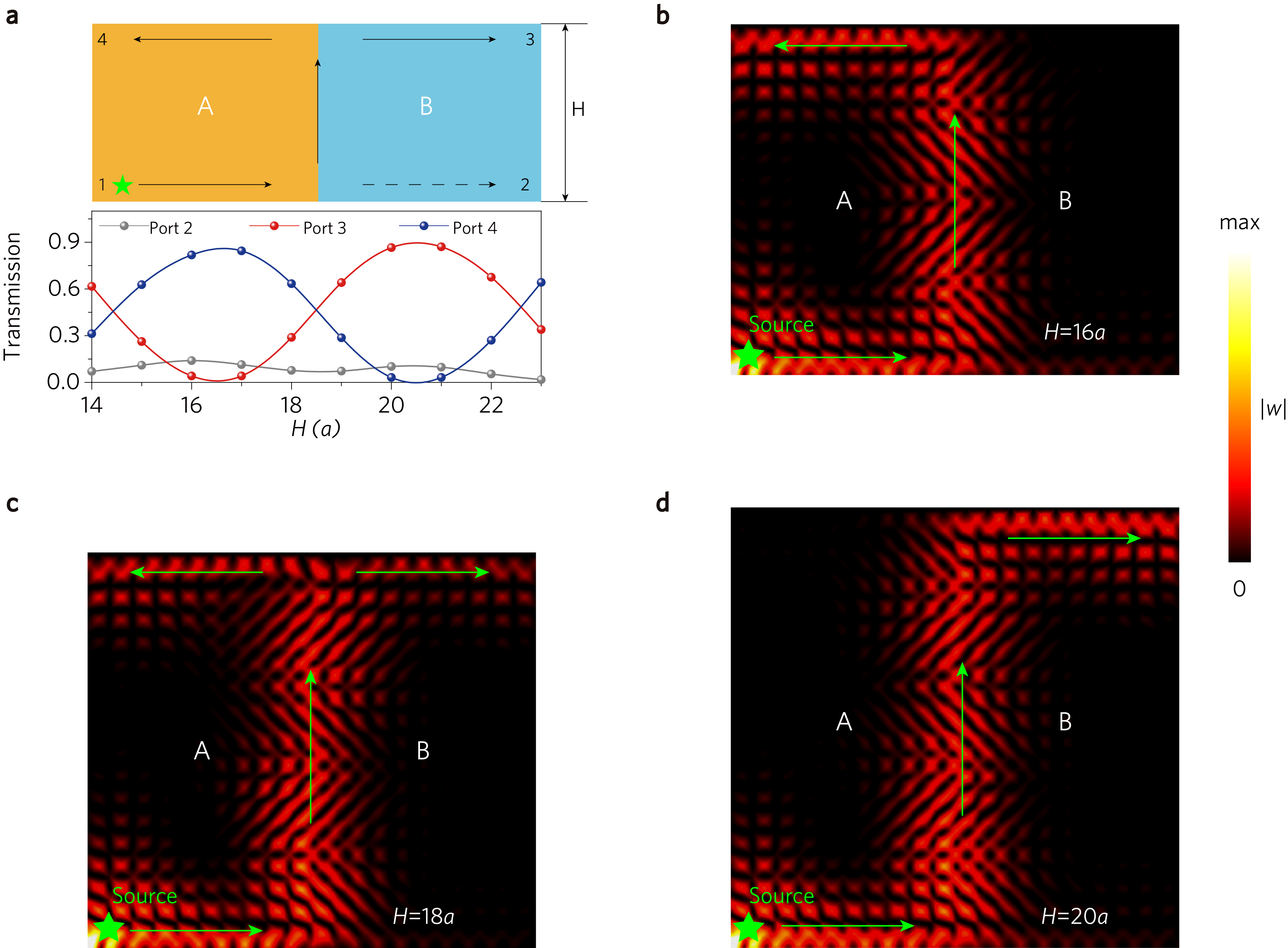


**Supplementary Figure 11: Selective transport of elastic waves for the clamped boundaries.** **a**, The same configuration with that based on free boundaries. The up and down boundaries of the device are clamped. The lower panel shows the proportion of energy flux in each channel with respect to $H$. Excitation frequency is $26.75 kHz$. The gray, red, and blue curves represent the output port 2, 3, and 4, respectively. **b-d**, Simulation results of the out-of-plane displacement for three typical heights, namely $H=16a$, $18a$, and $20a$, respectively. The green circle denotes the source.

**Reference**

1. Lu, J., Qiu, C., Deng, W., Huang, X., Li, F., Zhang, F., & Liu, Z. Valley topological phases in bilayer sonic crystals. *Phys. Rev. Lett.* **120**, 116802 (2018).
2. Yu, R., Qi, X. L., Bernevig, A., Fang, Z., & Dai, X. Equivalent expression of Z_2_ topological invariant for band insulators using the non-Abelian Berry connection. *Phys. Rev. B* **84**, 075119 (2011).
3. Süsstrunk, R., & Huber, S. D. Classification of topological phonons in linear mechanical metamaterials. *P. Natl. Acad. Sci. USA* **113**, E4767-E4775 (2016).
4. Duda, T., & Raghavan, L. V. 3D metal printing technology. *IFAC-Papers On Line* **49**, 103-110 (2016).
5. Zhang, X., Wang, H. X., Lin, Z. K., Tian, Y., Xie, B., Lu, M. H., & Jiang, J. H. Second-order topology and multidimensional topological transitions in sonic crystals. *Nat. Phys.* **15**, 582-588 (2019).
